# Cognitive phenotype of juvenile absence epilepsy: An investigation of patients and unaffected siblings

**DOI:** 10.1111/epi.17719

**Published:** 2023-08-10

**Authors:** Lorenzo Caciagli, Corey Ratcliffe, Fenglai Xiao, Louis A. van Graan, Karin Trimmel, Christian Vollmar, Maria Centeno, John S. Duncan, Pamela J. Thompson, Sallie Baxendale, Matthias J. Koepp, Britta Wandschneider

**Affiliations:** ^1^ Department of Clinical and Experimental Epilepsy UCL Queen Square Institute of Neurology London UK; ^2^ MRI Unit Epilepsy Society Buckinghamshire UK; ^3^ Department of Neurology, Inselspital, Sleep‐Wake‐Epilepsy‐Center, Bern University Hospital University of Bern Bern Switzerland; ^4^ Department of Pharmacology and Therapeutics, Institute of Systems, Molecular, and Integrative Biology University of Liverpool Liverpool UK; ^5^ Department of Neuroimaging and Interventional Radiology National Institute of Mental Health and Neurosciences Bangalore India; ^6^ Department of Neurology Medical University of Vienna Vienna Austria; ^7^ Department of Neurology Ludwig‐Maximilians‐Universität Munich Germany; ^8^ Epilepsy Unit, Department of Neurology Hospital Clínic de Barcelona Barcelona Spain

**Keywords:** cognition, endophenotype, juvenile absence epilepsy, juvenile myoclonic epilepsy, neuropsychology

## Abstract

**Objective:**

The cognitive profile of juvenile absence epilepsy (JAE) remains largely uncharacterized. This study aimed to: (1) elucidate the neuropsychological profile of JAE; (2) identify familial cognitive traits by investigating unaffected JAE siblings; (3) establish the clinical meaningfulness of JAE‐associated cognitive traits; (4) determine whether cognitive traits across the idiopathic generalized epilepsy (IGE) spectrum are shared or syndrome‐specific, by comparing JAE to juvenile myoclonic epilepsy (JME); and (5) identify relationships between cognitive abilities and clinical characteristics.

**Methods:**

We investigated 123 participants—23 patients with JAE, 16 unaffected siblings of JAE patients, 45 healthy controls, and 39 patients with JME—who underwent a comprehensive neuropsychological test battery including measures within four cognitive domains: attention/psychomotor speed, language, memory, and executive function. We correlated clinical measures with cognitive performance data to decode effects of age at onset and duration of epilepsy.

**Results:**

Cognitive performance in individuals with JAE was reduced compared to controls across attention/psychomotor speed, language, and executive function domains; those with ongoing seizures additionally showed lower memory scores. Patients with JAE and their unaffected siblings had similar language impairment compared to controls. Individuals with JME had worse response inhibition than those with JAE. Across all patients, those with older age at onset had better attention/psychomotor speed performance.

**Significance:**

JAE is associated with wide‐ranging cognitive difficulties that encompass domains reliant on frontal lobe processing, including language, attention, and executive function. JAE siblings share impairment with patients on linguistic measures, indicative of a familial trait. Executive function subdomains may be differentially affected across the IGE spectrum. Cognitive abilities are detrimentally modulated by an early age at seizure onset.

**Key Points**

- JAE presents with multidomain cognitive impairment involving language, attention/psychomotor speed, and executive function
- Impaired language is common to people with JAE and their unaffected siblings, suggestive of a familial trait (endophenotype)
- Response inhibition is worse in JME than JAE, indicating partially distinct cognitive profiles across the IGE spectrum
- Early age at epilepsy onset is associated with worse attentional difficulties in JAE and JME

## 1 INTRODUCTION

Juvenile absence epilepsy (JAE) is an idiopathic generalized epilepsy (IGE) syndrome,^1^ and typically presents with onset of absence seizures in late childhood or adolescence. Most patients also experience generalized tonic–clonic seizures.^2^ JAE is assumed to be polygenetic in origin, similar to the other three IGE syndromes (childhood absence epilepsy [CAE], juvenile myoclonic epilepsy [JME], and generalized tonic–clonic seizures alone).^3^ Seizure onset in JAE and JME coincides with a crucial phase of neurodevelopment.^4^ It is hypothesized that alterations in developmental trajectories in JAE and JME may also lead to impaired cognition.^5^, ^6^, ^7^

Cognitive comorbidities are increasingly recognized as part of the IGE phenotype,^8^, ^9^ can predate seizure onset by several years,^10^, ^11^ and persist after seizure control is achieved.^9^, ^12^ Cognitive difficulties have also been reported in seizure‐unaffected first‐degree relatives of patients with IGE and JME, the most common IGE syndrome.^13^, ^14^, ^15^ These cognitive traits are interpreted as intermediate phenotypes or *endophenotypes*, that is, disease signatures that are more prevalent in patients and first‐degree relatives than the general population, are closely related to the underlying genotype,^16^ and allow differentiating the familial underpinnings of cognitive traits from the effects of disease activity or antiseizure medication (ASM).

Cognitive studies in absence epilepsies have focused mainly on CAE or combined CAE and JAE cohorts, given the similarities in disease pathological mechanisms^17^ and clinical presentation,^2^ and revealed lower intelligence quotient (IQ) along with impairment of visual–spatial processing, attention, language, and executive function.^8^, ^18^, ^19^, ^20^, ^21^ However, investigations that detail the cognitive profile of JAE, address the underlying familial determinants, and probe the syndrome specificity of cognitive traits are limited.

Here, we aimed to characterize the cognitive phenotype of a homogeneous, well‐defined JAE cohort via a comprehensive neuropsychological test battery. We also investigated unaffected siblings of JAE patients, to identify familial cognitive traits (JAE endophenotypes). We addressed the clinical meaningfulness of cognitive phenotypes in JAE by contextualizing our findings against population reference means. Then, we directly compared individuals with JAE and JME, to highlight syndrome‐specific and shared traits, and provide further insights into the presumed overlap of cognitive comorbidities across the IGE spectrum.^8^ Finally, we assessed the relationship between cognitive performance and clinical characteristics, such as age at onset and disease duration.

## 2 MATERIALS AND METHODS

### 2.1 Participants

In this prospective cross‐sectional study, we investigated 123 consecutively recruited participants: 23 JAE patients, 16 seizure‐unaffected siblings of 11 index patients with JAE, 39 JME patients, and 45 healthy control participants with no family history of epilepsy or other neurological disorders. All patients were recruited from epilepsy outpatient clinics at the National Hospital for Neurology and Neurosurgery (London, UK) and the Chalfont Centre for Epilepsy (Buckinghamshire, UK), between 2007 and 2019. Controls were recruited from local communities in North West London and Chalfont St. Peter, UK.

Individuals with JAE had a typical clinical presentation, with age at onset in late childhood or early puberty (median = 12 years, interquartile range = 6). All had absence seizures, and 83% had a history of generalized tonic–clonic seizures (GTCS). Three patients (13%) reported infrequent myoclonus associated with absence seizures, which is compatible with a JAE diagnosis.^2^ Sixteen patients (70%) had seizures in the year before the study, all of whom had absences and six of whom also had GTCS. Family history of epilepsy was confirmed in 12 patients (52%). All patients with JAE had a typical routine electroencephalogram (EEG) with interictal 3–4‐Hz generalized spike–wave discharges; seven (30%) had been seizure‐free for at least 1 year prior to the investigation. No JAE sibling had ever experienced seizures, except for one individual who had one clearly provoked GTCS episode following a head trauma during a motor vehicle accident. All patients with JME had myoclonic seizures and GTCS; 14 of 39 patients (36%) had absences. All individuals with JME had a typical EEG with interictal generalized polyspike‐and‐wave discharges; 20 of them (51%) had been seizure‐free for at least 1 year. Clinical magnetic resonance imaging (MRI) was normal in all participants. We previously reported on some cognitive results of people with JME and part of the controls.^5^, ^22^

Patients with JAE, their siblings, and controls had comparable age. Patients with JME were older than those with JAE and controls. Groups were comparable for sex. Patients with JAE and their siblings had lower levels of education than controls. Related statistics, including further demographics and clinical details, are provided in Table 1.

**TABLE 1:** Demographic data, clinical characteristics, and questionnaires.

Characteristic	CTR, *n* = 45	JAE, *n* = 23	SIB, *n* = 16	JME, *n* = 39	Test statistic	*p*	Post hoc tests, Bonferroni corrected
Age, years, mean (SD)	28.4 (6.6)	24.4 (6.6)	26.0 (8.0)	34.3 (10.7)	*F* = 8.4	**<.001**	JAE vs. CTR: .380 SIB vs. CTR: 1.000 JAE vs. SIB: 1.000 **JME vs. JAE: <.001** **JME vs. CTR: .009**
Sex, F/M	29/16	16/7	5/11	22/17	FET = 6.6	.079	N/A
Education, ordinal, median (IQR)	3.0 (1.5)	2.0 (2.0)	2.0 (1.8)	3.0 (1.0)	*H* = 20.7	**<.001**	**JAE vs. CTR: .001** **SIB vs. CTR: .001** JAE vs. SIB: 1.00 JME vs. JAE: .834 JME vs. CTR: .154
Dysexecutive traits (DEX), median (IQR)	14.0 (9.5)	27.5 (14.8)	10.0 (14.5)	‐^a^	*H* = 17.3	**<.001**	**JAE vs. CTR: .008** SIB vs. CTR: 1.000 **JAE vs. SIB: .010**
Anxiety (HADS‐A), median (IQR)	5.0 (5.0)	5.0 (6.0)	2.5 (3.8)	6.0 (5.5)	*H* = 17.0	**<.001**	JAE vs. CTR: .282 SIB vs. CTR: .255 **JAE vs. SIB: .004** JME vs. JAE: 1.00 JME vs. CTR: .230
Depression (HADS‐D), median (IQR)	1.0 (2.0)	2.0 (3.0)	0.0 (2.8)	2.0 (4.3)	*H* = 18.0	**<.001**	**JAE vs. CTR: .021** SIB vs. CTR: 1.000 **JAE vs. SIB: .030** JME vs. JAE: 1.00 JME vs. CTR: **.009**
ASM, median (IQR)	N/A	2.0 (1.0)	N/A	2.0 (1.0)	*H* = .008 (JME vs. JAE)	.927	
Sodium valproate, yes/no	N/A	10/23	N/A	24/15	FET = 1.9 (JME vs. JAE)	.195	
Topiramate or zonisamide, yes/no	N/A	2/21	N/A	3/36	FET = .02 (JME vs. JAE)	.619	
Disease duration, years, median (IQR)	N/A	11.3 (11.0)	N/A	16.0 (18.0)	*H* = 5.9 (JME vs. JAE)	.**015**	
Age at onset, years, median (IQR)	‐	12.0 (6.0)	‐	15.0 (4.0)	*H* = 9.3 (JME vs. JAE)	**.002**	

*Note:* Bold indicates statistical significance.

Abbreviations: ASM, anti‐seizure medication(s); CTR, controls; DEX, Dysexecutive Questionnaire; F, female; FET, Fisher exact test statistic; HADS‐A, Hospital Anxiety and Depression Scale–Anxiety; HADS‐D, Hospital Anxiety and Depression Scale–Depression; IQR, interquartile range; JAE, juvenile absence epilepsy; JME, juvenile myoclonic epilepsy; M, male; N/A, not applicable; SIB, unaffected siblings of patients with JAE.

^a^ Not provided, as only available in five individuals with JME owing to study protocol changes.

### 2.2 Standard protocol approvals, registrations, and patient consents

Participant recruitment received ethical approval from the University College London and University College London Hospitals Joint Research Ethics Committee (06/N059 and 11/LO/0439). Written informed consent was obtained from all participants in accordance with the standards of the Declaration of Helsinki.

### 2.3 Self‐assessment questionnaires

We used the Hospital Anxiety and Depression Scale (HADS), a self‐assessment questionnaire, to address current symptoms of anxiety (HADS‐A) and low mood (HADS‐D).^23^ Participants also completed the Dysexecutive Questionnaire, which measures everyday life problems resulting from dysexecutive traits.^24^

### 2.4 Neuropsychological tests and cognitive domains

All participants underwent a comprehensive neuropsychological test battery, as described elsewhere,^8^ whose completion required about 90 min with standardized interspersed breaks. This battery provides measures of estimated IQ (National Adult Reading Test)^25^ and addresses four cognitive domains:

1. *Attention/psychomotor speed*: Trail Making Test (TMT) Part A,^26^ Stroop Color–Word Test, C and W scores.^27^
2. *Language*: Vocabulary and Similarities on the Wechsler Adult Intelligence Scale (WAIS), third edition,^28^ letter and category fluency,^8^, ^29^ and visual confrontation naming.^30^
3. *Memory*: Verbal and visual learning and recall on the Adult Memory and Information Processing Battery.^31^
4. *Executive function*: Digit Span and Mental Arithmetic (WAIS),^28^ interference measure of the Stroop Test,^27^ and TMT B‐A score (Task Switching).^26^

Test details are provided in Table S1. Letter fluency and similarities can also be regarded as executive function tests.^32^ Across all our participants, however, both letter fluency and similarities had higher correlations with language than with executive function measures (Supplementary Methods), and were thus considered as language measures.^33^

To reduce data dimensionality,^5^ we ran principal component analyses (PCAs) on the scores of tests subsumed under a given cognitive domain (attention/psychomotor speed, language, memory, executive function) across all individuals. For each of the four PCAs, we verified that the first principal component had an eigenvalue > 1 and retained the first component for each cognitive domain except for executive function, for which two components were retained: a global component that mirrored performance across all four tests (executive function–global) and a second component with values almost exclusively driven by response inhibition scores (executive function–response inhibition). Tables S2–S5 provide correlation matrices for tests subsumed under a given domain and loadings for each component. Thus, our subsequent analyses compared groups in relation to five principal component scores, that is, *cognitive domain scores*.

### 2.5 Statistical analyses: Demographics, clinical data, and questionnaires

Data were analyzed using IBM SPSS v28 and R 4.2.0. For analysis of demographic and clinical data, we used analysis of variance, Kruskal–Wallis, and Fisher exact tests for continuous parametric, nonparametric, and categorical data, respectively; post hoc tests were Bonferroni‐corrected for multiple comparisons. Kruskal–Wallis and Fisher exact test were used to compare clinical parameters between individuals with JAE and JME. Some data for education, questionnaires, and some cognitive tests were missing because of slight changes in the study protocol. Thus, we used Little's missing completely at random^34^ on all cases, all neuropsychological test measures, and all education, anxiety, depression, and dysexecutive trait questionnaires, which showed no association between data missingness and any values (*χ* ^2^ = 506.8, *df* = 480, *p* = .19).

### 2.6 Statistical analyses: Group comparisons, JAE patients, siblings, and controls, Aims 1–2

We used multivariate analysis of covariance (MANCOVA) to identify global differences in cognitive domain scores among groups. We included age and (binary) sex as covariates in all analyses and used Wilk's lambda (*λ*) as multivariate test statistic. To address our study aims, we envisioned the following models:

1. Comparison of individuals with JAE, their unaffected siblings, and controls, to ascertain the cognitive profile of JAE (Aim 1) and to identify familial cognitive traits, that is, JAE endophenotypes (Aim 2).
2. Sensitivity analyses for Aims 1 and 2. We reran models specified in (1) with additional covariates, represented by features that differed across groups: education (first sensitivity analysis), and depression and anxiety (second sensitivity analysis).

To test for group differences in individual cognitive domains, we then performed univariate analyses using analyses of covariance (ANCOVAs), as previously described,^5^, ^15^ and compared JAE patients, JAE siblings, and controls. For these five “domain‐wise” ANCOVAs, we used age and sex as covariates, and adjusted *p*‐values of each test for multiple comparisons via the false discovery rate (FDR) procedure; all post hoc pairwise tests were Bonferroni‐corrected. Cohen's *d* was used as a measure of effect size and was computed on residualized metrics after adjusting for age and sex via multiple regression. In all illustrations, cognitive domain scores are plotted as raw, whereas the shown *p*‐values refer to age‐ and sex‐adjusted statistics. Missing data were addressed via pairwise deletion in all analyses.

### 2.7 Statistical analyses: Contextualization of JAE findings against test norms, Aim 3

Follow‐up analyses using neuropsychological test norms contextualized statistically significant group differences between JAE and controls from a clinical standpoint. Where possible, we used recently published norms drawn from culturally and geographically representative populations (Data S1). As different from analyses investigating group differences across cognitive domains, we conducted norm‐based analyses testwise. In the case that no suitable norms were available for a specific measure (e.g., Trail Making B‐A), we considered the most similar surrogate norm (e.g., Trail Making B), and proceeded after verifying via *z*‐tests (Data S1) that effect sizes (Cohen's *d*) for the comparison of individuals with JAE and controls in relation to the original and substitute measure were comparable.

For each test measure, scores of individuals with JAE were converted into *z‐*scores according to the formula: *z*_JAE_ = (score_JAE_ − *μ*)/*σ*, where *μ*/*σ* corresponds to the mean/SD of a given norm. For each normalized test score, group‐level deviations of patients' *z*‐scores from zero were assessed with two‐tailed, one‐sample *t‐*tests. To provide a qualitative judgment regarding clinical meaningfulness, group performance on a given cognitive test was considered impaired if the mean in JAE was ≥1 SD below the normative mean.

### 2.8 Statistical analyses: Group comparisons, JAE versus JME, Aim 4

MANCOVA and domainwise ANCOVAs comparing JME and JAE were conducted using the same rationale and methodology described for Aims 1–2. As two groups were compared, no post hoc tests were necessary. For completeness and to ascertain representativity of the cognitive profile of our JME sample compared to prior work, we also compared performance of individuals with JME and healthy controls (Supplementary Results).

### 2.9 Statistical analyses: Role of clinical characteristics, Aim 5

To investigate the potential influence of disease severity, we compared the JAE subgroup with uncontrolled seizures and healthy controls, using the same methodology described for Aim 1. To probe the influence of timing of disease onset on cognition, we conducted correlation analyses of cognitive domain scores with age at onset across all patients. We also conducted correlation analyses of cognitive domain scores with disease duration as a marker of disease chronicity. These correlations were restricted to domains for which there were significant differences between JAE and controls, or between JAE and JME. As the correlation between age at onset and disease duration approached statistical significance (*ρ* = −.24, *p* = .068), we opted for partial correlations of domain scores with age at onset, covaried for duration, and vice versa. Chronological age and age at onset were not significantly correlated (*ρ* = −.10, *p* = .46).

## 3 RESULTS

### 3.1 Demographic and clinical data and self‐assessment questionnaires

Complete statistical details are provided in Table 1. Patients with JAE reported more symptoms of depression than their siblings and controls (*p*_Bonferroni_ = .030/.021, respectively), and more symptoms of anxiety than their siblings (*p*_Bonferroni_ = .004). Median scores for anxiety and depression symptoms were largely below the cutoff scores used to define mild symptoms in all groups.^23^ Self‐reported dysexecutive traits were more pronounced in individuals with JAE than in siblings and controls (*p*_Bonferroni_ = .010/.008, respectively). Patient groups were comparable for ASM number, proportion of patients treated with sodium valproate, and proportion of patients treated with topiramate or zonisamide. Patients with JAE had younger age at onset and shorter disease duration than those with JME (*p* = .002/.015, respectively).

### 3.2 Aims 1 and 2: Cognitive performance in JAE and JAE siblings compared to controls

**FIGURE 1:**
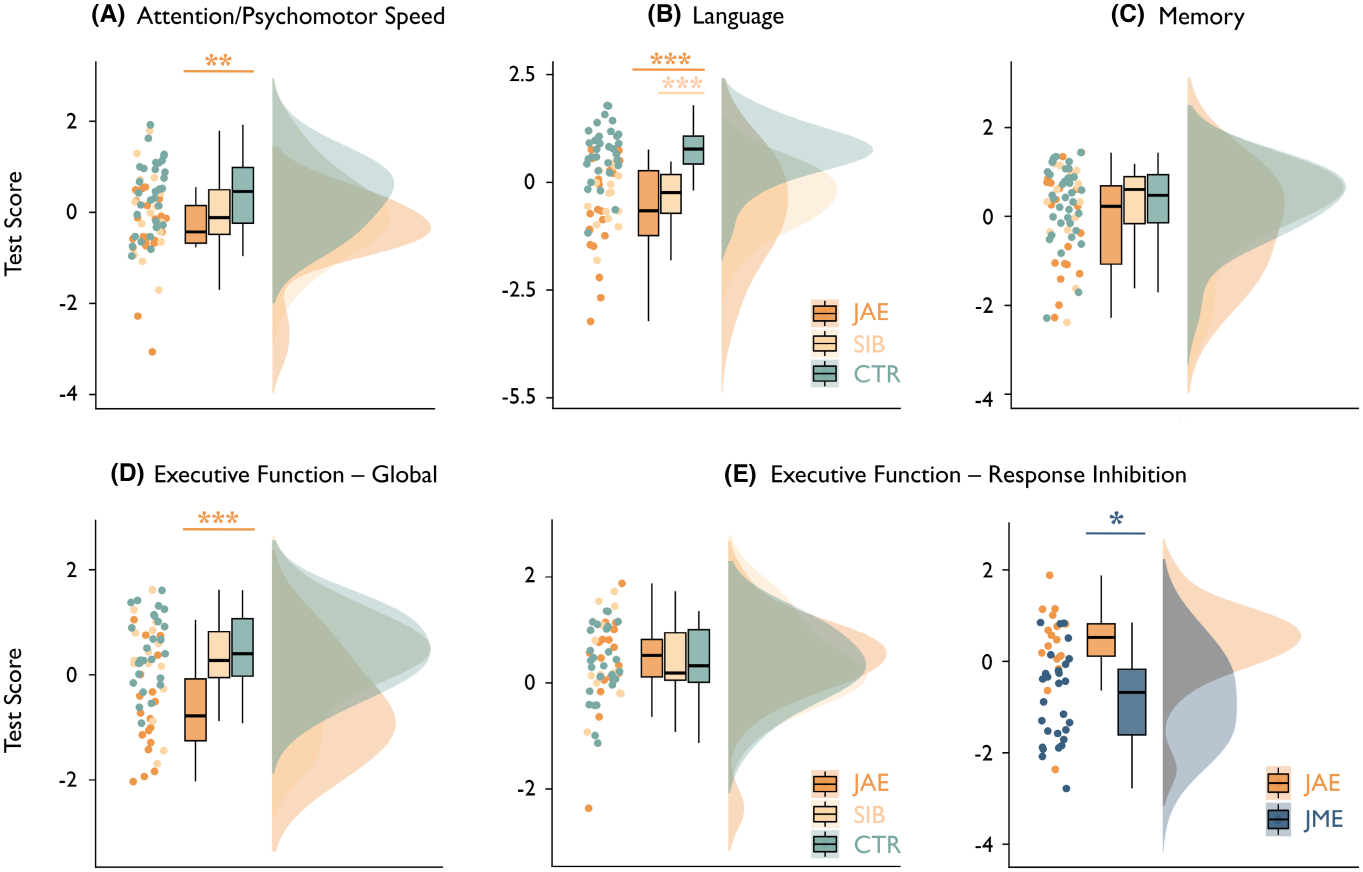
Group comparisons: cognitive domain scores. (A–D) Data in individuals with juvenile absence epilepsy (JAE), unaffected JAE siblings (SIB), and controls (CTR) for cognitive domain scores pertaining to attention/psychomotor speed (A), language (B), memory (C), and global executive function (D). (E) Data for the response inhibition‐weighted executive function domain; the plot on the left‐hand side of the panel refers to the comparison of individuals with JAE, their unaffected siblings, and healthy controls, whereas the plot on the right‐hand side refers to the comparison of individuals with JAE against those with juvenile myoclonic epilepsy (JME). For each measure, we used open‐source code to generate *raincloud* plots (https://github.com/RainCloudPlots/RainCloudPlots), and show a combination of single datapoints, boxplots, and probability distributions. Statistical details are reported in Tables 2 and 3 and in the main text. In (A–D), asterisks refer to *p‐*values for Bonferroni‐corrected, age‐ and sex‐adjusted post hoc tests of the analyses of covariance (ANCOVAs; JAE vs. controls, indicated by underlying orange bars; siblings vs. controls, indicated by underlying sunset [light orange] bars). In E, the asterisk refers to an ANCOVA *p‐*value (JME vs. JAE, indicated by blue bars) adjusted for the false discovery rate (i.e., across cognitive domains). Statistical details are provided in the main text. *p < .05, corrected; **p < .01, corrected; ***p < .001, corrected.

MANCOVA yielded a significant effect of group on cognitive performance (Wilk's *λ* = .51, *F*_10, 90_ = 3.6, *p* = .0005). ANCOVAs showed significant group differences across multiple cognitive domains, including attention/psychomotor speed, language, and global executive function (all *p*_FDR_ < .01; Table 2, Figure 1). Post hoc tests showed worse performance in individuals with JAE than controls for the above three domains (all *p*_Bonferroni_ ≤ .01; Cohen's *d* range = −1.45 to −.99). Language impairment was common to individuals with JAE and their siblings (*p*_Bonferroni_ ≤ .001 vs. controls, *d* = −1.45 and −1.59 for JAE and JAE siblings, respectively). There were no differences in estimated IQ among individuals with JAE, JAE siblings, and controls (Table 2, Figure S1). JAE siblings had an intermediate position between patients and controls for attention/psychomotor speed and global executive function, with no statistically significant differences against either group.

**TABLE 2:** Comparison of JAE, JAE siblings, and controls.

	Effect of group, *F* statistic	*p*_FDR_ (uncorrected *p*)	Mean (SD)	Post hoc *p*, Bonferroni corrected	Effect size, Cohen's *d*
Estimated IQ, NART	*F*_2, 75_ = 2.8	.084 (.070)	JAE: 103.4 (8.5) SIB: 106.4 (4.8) CTR: 108.8 (7.7)		JAE vs. CTR: −.56 SIB vs. CTR: −.29 JAE vs. SIB: −33
Attention/psychomotor speed	*F*_2, 63_ = 6.0	**.008 (.004)**	JAE: −.43 (.87) SIB: −.02 (.89) CTR: .40 (.76)	JAE vs. CTR: **.003** SIB vs. CTR: .67 JAE vs. SIB: .28	JAE vs. CTR: **−.99** SIB vs. CTR: −.41 JAE vs. SIB: −.56
Language	*F*_2, 65_ = 18.3	**<.0001 (<.0001)**	JAE: −.65 (1.15) SIB: −.34 (.64) CTR: .69 (.65)	JAE vs. CTR: **<.001** SIB vs. CTR: **<.001** JAE vs. SIB: 1.0	JAE vs. CTR: **−1.45** SIB vs. CTR: **−1.59** JAE vs. SIB: −.21
Memory	*F*_2, 66_ = 3.6	.052 (.034)	JAE: −.21 (1.13) SIB: .21 (1.0) CTR: .34 (.84)		JAE vs. CTR: −.76 SIB vs. CTR: −.35 JAE vs. SIB: −.38
Executive function–global	*F*_2, 55_ = 9.8	**.0007 (.0002)**	JAE: −.64 (.94) SIB: .21 (.94) CTR: .47 (.69)	JAE vs. CTR: **<.001** SIB vs. CTR: .30 JAE vs. SIB: .12	JAE vs. CTR: **−1.39** SIB vs. CTR: −.59 JAE vs. SIB: −.72
Executive function–response inhibition	*F*_2, 55_ = 1.0	.933 (.933)	JAE: .38 (.90) SIB: .42 (.74) CTR: .35 (.68)		JAE vs. CTR: .02 SIB vs. CTR: −.10 JAE vs. SIB: .11

*Note*: Multivariate model: Wilk's λ = .51, *F*_10, 90_ = 3.6, p = .0005. All statistical analyses controlled for age and sex. Bold indicates statistical significance.

Abbreviations: CTR, controls; FDR, false discovery rate; IQ, intelligence quotient; JAE, juvenile absence epilepsy; NART, National Adult Reading Test; SIB, unaffected JAE siblings.

### 3.3 Aims 1 and 2: Sensitivity analyses

Repeat MANCOVA covarying for education in addition to age and sex confirmed a significant effect of group on cognitive performance (*p* = .006); repeat MANCOVA covarying for self‐reported anxiety and depression symptoms in addition to age and sex yielded a significant effect of group on cognitive performance (*p* = .007). For both these MANCOVAs, there were no substantial changes in statistical significance and effect size for the above‐described group differences (Supplementary Results).

### 3.4 Aim 3: Norm‐based analyses

Analysis of *z*‐scores adjusted based on population reference means corroborated a lower performance of individuals with JAE on attention/psychomotor speed (TMT Part A, Stroop–Words), language (visual confrontation naming, letter fluency), and executive function (TMT Part B) tests (all *p*_FDR_ < .01, one‐sample *t‐*tests; Table 3). Cognitive impairment in JAE could be regarded as clinically meaningful (<1 SD below the normative mean) for one test in each of the above domains (TMT Part A, naming, TMT Part B, *z*‐score: 1.37/1.98/1.94, respectively); notably, scores for one language and one executive function test were 1.5 SD lower than the normative mean. Letter fluency and Stroop–Word approached clinical meaningfulness (*z*‐score: −.81/−.78, respectively).

**TABLE 3:** Performance in JAE compared to population reference means.

Test	JAE, *z*‐score mean (SD)	Effect of group, *T*‐statistic	*p*_FDR_ (uncorrected *p*)
Attention/psychomotor speed			
Trail Making Test–Part A^a^	**−1.37** (1.56)	*t*_1, 21_ = −4.1	**.0013 (.0005)**
Stroop–Words, items in 45 s	**−.78** (.88)	*t*_1, 20_ = −4.0	**.0013 (.0006)**
Stroop–Color, items in 45 s	.10 (.99)	*t*_1, 20_ = .5	.794 (.650)
Language			
Vocabulary	.31 (.90)	*t*_1, 20_ = 1.6	.200 (.123)
Similarities	.10 (.82)	*t*_1, 20_ = .5	.794 (.599)
McKenna graded naming	**−1.98** (1.12)	*t*_1, 20_ = −8.3	**<.0001 (<.0001)**
Phonemic fluency, letter S	**−.81** (.78)	*t*_1, 21_ = −4.8	**.0003 (<.0001)**
Semantic fluency, category: animals	−.35 (1.05)	*t*_1, 21_ = −1.6	.200 (.127)
Executive function			
Digit span	−.07 (1.18)	*t*_1, 18_ = −.3	.879 (.799)
Arithmetic	.01 (.34)	*t*_1, 18_ = .1	.941 (.941)
Trail Making Test–Part B^a^	**−1.94** (1.49)	*t*_1, 21_ = 6.1	**<.0001 (<.0001)**

*Note*: The table shows scores normalized based on population reference means for tests belonging to cognitive domains that differed between individuals with JAE and controls (attention/psychomotor speed, language, executive function). Bold indicates statistical significance.

Abbreviations: FDR, false discovery rate; JAE, juvenile absence epilepsy.

^a^ For Trail Making Test, Parts A and B, larger scores signify worse performance. To homogenize reporting with that for the other tests and to facilitate interpretability, *z*‐scores in this table were multiplied by −1.

### 3.5 Aim 4: Cognitive performance in JAE versus JME

MANCOVA showed no overall group differences in cognitive performance (Wilk's *λ* = .78, *F*_5, 36_ = 2.9, *p* = .104). Domainwise ANCOVAs showed better performance on the executive function, response inhibition‐weighted domain in JAE compared to JME (*p*_FDR_ = .023, *d* = .79; Table 4, Figure 1). Details regarding the comparison of people with JME and controls are provided in Data S1 (Supplementary Results, Table S6).

**TABLE 4:** Comparison of JAE and JME.

	Effect of group, *F* statistic	*p*_FDR_ (uncorrected *p*)	Mean (SD)	Effect size, Cohen's *d*
Estimated IQ, NART	*F*_1, 54_ = 2.1	.442 (.149)	JAE: 103.4 (8.5) JME: 108.4 (10.9)	JAE vs. JME: −.35
Attention/psychomotor speed	*F*_1, 52_ = 1.8	.442 (.221)	JAE: −.43 (.87) JME: −.08 (1.2)	JAE vs. JME: −.31
Language	*F*_1, 53_ = .3	.694 (.578)	JAE: −.65 (1.15) JME: −.11 (.96)	JAE vs. JME: −.14
Memory	*F*_1, 54_ = .4	.694 (.534)	JAE: −.21 (1.13) JME: −.29 (.98)	JAE vs. JME: −.16
Executive function–global	*F*_1, 42_ = .03	.876 (.876)	JAE: −.64 (.94) JME: −.15 (1.1)	JAE vs. JME: −.04
Executive function–response inhibition	*F*_1, 42_ = 9.4	**.023 (.004)**	JAE: .38 (.90) JME: −.81 (1.00)	JAE vs. JME: **.79**

*Note*: Multivariate model: Wilks's λ = 78, *F*_5, 36_ = 2.9, p = .104. All statistical analyses controlled for age and sex. Bold indicates statistical significance.

Abbreviations: FDR, false discovery rate; IQ, intelligence quotient; JAE, juvenile absence epilepsy; JME, juvenile myoclonic epilepsy; NART, National Adult Reading Test.

### 3.6 Aim 5: Role of clinical characteristics

MANCOVA comparing JAE patients with ongoing seizures against controls showed similar effects as in the main JAE analysis (Wilk's *λ* = .47, *F*_5, 26_ = 5.8, *p* = .001; Table 5). ANCOVAs showed weaknesses in attention/psychomotor speed, language, and global executive function, with similar effect sizes as those for comparisons of the whole JAE group and controls (all *p*_FDR_ < .01, *d* range = −1.54 to −1.19). As distinct from the whole JAE group, JAE individuals with ongoing seizures had significantly reduced memory performance compared to controls (*p*_FDR_ = .002, *d* = −1.11).

**TABLE 5:** Comparison of JAE patients with ongoing seizures and controls.

	Effect of group, *F* statistic	*p*_FDR_ (uncorrected *p*)	Mean (SD)	Effect size, Cohen's *d*
Estimated IQ, NART	*F*_1, 53_ = 4.3	.052 (.043)	JAE sz: 103.1 (9.0) CTR: 108.8 (7.7)	JAE sz vs. CTR: −.60
Attention/psychomotor speed	*F*_1, 43_ = 13.5	**.0014 (.0007)**	JAE sz: −.43 (.88) CTR: .40 (.76)	JAE sz vs. CTR: **−1.19**
Language	*F*_1, 43_ = 22.7	**.0001 (<.0001)**	JAE sz: −.58 (1.12) CTR: .69 (.65)	JAE sz vs. CTR: **−1.52**
Memory	*F*_1, 46_ = 11.6	**.002 (.0013)**	JAE sz: −.48 (1.23) CTR: .34 (.84)	JAE sz vs. CTR: **−1.11**
Executive function–global	*F*_1, 35_ = 19.3	**.0003 (.0001)**	JAE sz: −.65 (.99) CTR: .47 (.69)	JAE sz vs. CTR: **−1.54**
Executive function–response inhibition	*F*_1, 35_ = .002	.962 (.962)	JAE sz: .30 (.99) CTR: .35 (.68)	JAE sz vs. CTR: −.02

*Note*: Multivariate model: Wilk's λ = .47, *F*_5, 26_ = 5.8, p = .001. All statistical analyses controlled for age and sex. Bold indicates statistical significance.

Abbreviations: CTR, controls; FDR, false discovery rate; IQ, intelligence quotient; JAE, juvenile absence epilepsy; NART, National Adult Reading Test; sz, with seizures.

Controlling for disease duration, we found significant correlations of age at onset with attention/psychomotor speed, executive function–response inhibition (*ρ* = .33, *p*_FDR_ = .031; and *ρ* = −.43, *p*_FDR_ = .015, respectively, adjusted for number of correlations), and executive function–global at an uncorrected threshold (*ρ* = .31, *p* = .046, *p*_FDR_ = .061). Later age at onset was associated with better attention/psychomotor speed and global executive function, but worse response inhibition. The correlation of language with age at onset was not statistically significant (*ρ* = .13, *p* = .36).

Controlling for age at onset, we found significant correlations of duration of disease with executive function–response inhibition (*ρ* = −.42, *p*_FDR_ = .020), and with language at an uncorrected threshold (*ρ* = .29, *p* = .037, *p*_FDR_ = .074). The correlations of duration with attention/psychomotor speed and global executive function were not significant (*ρ* = −.01/.28, *p* = .97/.068, respectively).

## 4 DISCUSSION

Despite extensive investigation of JME, CAE, or mixed absence epilepsy cohorts,^8^, ^20^ detailed assessments of the cognitive profile of JAE are limited. Here, we characterized the cognitive phenotype of a homogeneous, well‐characterized JAE sample. Using an approach conceptually similar to previous literature, we adopted a dimensionality reduction method and focused on cognitive domains.^35^ We identified multidomain impairment in JAE that involved attention and psychomotor speed, language, and executive function. Unaffected JAE siblings had similar language impairment as patients and did not differ from patients or controls in the remainder domains. In keeping with previous work in IGE^14^ and in JME specifically,^13^, ^15^ our findings suggest that there is a familial component to the cognitive impairment in JAE. Comparison of JME and JAE showed an isolated difference in the form of poorer response inhibition in JME, suggesting some degree of syndrome specificity of executive function profiles. Similar to JAE, however, several cognitive domains were also affected in JME, consistent with a substantial overlap of cognitive profiles across IGE syndromes.^1^ Our sensitivity analyses excluded an influence of education and mood symptoms on the reported cognitive signatures. Correlation analyses corroborated the existence of discrete^35^ cognitive phenotypes, that is, cognitive profiles that are independent of epilepsy syndrome, and may rather be influenced by factors such as family history and neurodevelopment. Specifically, an early timing of disease onset and longer duration of disease adversely affected attention/psychomotor speed and executive function, indicating that neurodevelopmental alterations and disease chronicity may be important determinants of IGE‐associated cognitive difficulties.

In our JAE sample, estimated IQ was comparable to controls. Previous studies in mixed absence epilepsy reported lower IQ in patients than controls,^8^, ^20^ but we note that such scores fell within a range considered average for most patients.^8^ The effect sizes of performance differences between individuals with JAE and controls for attention/psychomotor speed, language, and executive function tests were large. Analyses contextualizing findings in JAE against population reference means showed impairment greater than 1 SD for one test in each of these domains, highlighting the clinical meaningfulness of cognitive difficulties in JAE.

We identified weaknesses in attention and psychomotor speed in JAE. Attentional difficulties are a key feature of mixed absence epilepsy samples.^8^, ^12^, ^20^ Prior work found attentional deficits in one third of their new onset CAE cohort, which persisted up to 20 weeks after treatment initiation, irrespective of seizure control.^12^ Collectively, attentional difficulties may be construed as a core characteristic of the cognitive phenotype of JAE, and absence epilepsy more broadly. Interestingly, the above authors also revealed subsequent detrimental effects of attentional deficits on long‐term memory, executive function, and academic achievement, and other research also pointed to attention as the necessary prerequisite for successful memory and other higher‐order abilities.^8^, ^12^ Thus, attentional difficulties likely represent an important driver of the multidomain cognitive impairment reported in our and prior studies. Prior combined EEG–functional MRI studies demonstrated altered activity patterns of large‐scale brain networks subserving attention, which appeared more prominent during preictal and ictal states.^21^, ^36^, ^37^ On balance, it is thus possible that attentional difficulties may also be modulated by disease activity.^17^, ^36^, ^37^

Similar to previous studies in mixed absence epilepsy,^20^ executive function was affected in our JAE sample. In addition, higher levels of self‐reported dysexecutive traits in people with JAE compared to their siblings and controls illustrate the tangible impact of such impairment on daily life. Executive dysfunction, however, was not homogeneous, with unaffected performance on a cognitive domain strongly defined by response inhibition scores. Executive function encompasses diverse subprocesses.^38^ Here, preserved response inhibition in JAE indicates the nonexclusive contingency of executive function on attention, prompting further research on the modulation of specific executive subdomains by attentional difficulties. Second, our findings confirm prior evidence of executive dysfunction in mixed IGE samples and in JME,^8^ highlighting commonalities along the IGE spectrum. We also found differences in response inhibition between JME and JAE patients, which indicates that executive function profiles may vary within the IGE spectrum. Our study cannot directly identify the underlying pathophysiology. We speculate that the genetic susceptibility to a specific IGE syndrome, and the associated differences in onset and clinical presentation, may interfere with different circuitry and stages of brain development, which could also influence the cognitive phenotypes. Importantly, response inhibition is elsewhere conceptualized as a marker of impulsivity and poor psychosocial outcome,^39^ which appears more prominent in JME than JAE. Our findings may thus have prognostic implications. We advocate replication with larger samples and more extensive executive function batteries.

Language was affected in the JAE group, in line with previous mixed absence epilepsy studies and meta‐analyses.^8^, ^20^, ^21^ Language abilities appear impaired across the whole epilepsy spectrum, particularly in syndromes with childhood onset. However, the severity of impairment appears slightly greater in absence epilepsies and temporal lobe epilepsy compared to JME and benign epilepsy with centrotemporal spikes.^40^ In our study, we identified similar language weaknesses in both people with JAE and their unaffected siblings. As IGEs have polygenetic etiology,^41^ investigating unaffected first‐degree relatives can identify intermediate phenotypes or *endophenotypes*, that is, traits that cosegregate in affected families, and help untangle familial contributions from other variables, such as disease duration or ASM.^16^ The linguistic domain represents a multidimensional construct, shaped by genetic and epigenetic determinants, socioeconomic factors, educational attainment, and other environmental factors.^42^ Thus, language impairment in JAE may be construed as a *familial* trait, that is, a trait that arises from the combination of genetic predisposition, sociocultural factors, and their interplay. In contrast, the more extensive cognitive difficulties seen in JAE patients than in their relatives, as previously documented for IGE and JME, may stem from the additional effects of disease burden, ASM, and other factors predisposing to recurrent seizures.^8^, ^13^, ^14^, ^15^ Thus, although the familial effect in JAE is limited to linguistic abilities, epilepsy itself and its associated factors appear to affect cognitive abilities in multiple domains.

Frequent seizures, in particular, can undermine cognitive function.^8^ Here, cognitive impairment in the subgroup of JAE patients with ongoing seizures overlapped with that of the whole JAE sample. Moreover, effect sizes for language measures with endophenotypic potential were nearly identical in the uncontrolled‐seizure subgroup, indicating a somewhat limited influence of clinical characteristics. Individuals with JAE and ongoing seizures, however, had worse memory performance, which relies on mesiotemporal processing. We speculate that neural networks underlying cognitive dysfunction may be broader in those with more severe disease, and more prominently encompass extrafrontal areas, which echoes recent evidence of mesiotemporal alterations in IGE syndromes.^5^, ^43^ We acknowledge, however, that the occurrence of subtle absence seizures in the seizure‐free subgroup cannot be completely excluded.

Imaging findings in CAE indicate abnormal frontotemporal cortical geometry^44^ and myelination,^45^ suggestive of abnormal neurodevelopment. The timing of disease onset, ranging from late childhood to early adolescence in JAE and JME, may lead to disruption of developmental trajectories in this critical phase, resulting in altered circuit maturation, abnormal cortical topography, and relatedly, cognitive impairment.^46^ In our study, older age at epilepsy onset was associated with (1) better performance on attention/psychomotor speed, (2) better global executive function performance at an uncorrected threshold, but (3) worse performance on response inhibition. These findings imply a complex interplay of epilepsy disease onset and developmental trajectories of slow‐maturing frontal networks,^47^ and the cognitive functions subserved by these. Although the association of greater attentional difficulties with an earlier disease onset is intuitive, we hypothesize that the opposite relationship between age at onset and response inhibition may be strongly determined by the performance of people with JME. These demonstrated poorer response inhibition compared to JAE and had a significantly older age at seizure onset.^1^ It is conceivable that patients with earlier disease onset could accumulate further injury to cognitive networks over time due to chronic disease. In our study, longer disease duration adversely affected response inhibition. However, the effect of age at onset on attention/psychomotor speed and global executive function was independent of duration, suggesting that some patterns of cognitive impairment in our patient cohort may rather be established during neurodevelopment. Our findings also align with prior observations by Hermann et al.,^35^ who reported cognitive phenotypes in childhood epilepsies that were influenced by factors linked to brain development, such as age at onset, and spanned different syndromes. Thus, we conclude that early seizures may be more universally harmful to the development of several cognitive networks, somewhat irrespective of syndromic classification.

Our study has limitations. Our sample size was relatively limited, which may affect generalizability. Further research using larger samples of patients and siblings is advocated to corroborate our findings and address the syndrome specificity of cognitive signatures along the IGE spectrum. We performed analyses based on test norms to investigate the clinical significance of our findings. However, appropriate norms were not available for some specific tests and some of the norms used were drawn from culturally and geographically different populations. Patients did not undergo simultaneous EEG monitoring during neuropsychological testing. Although cognitive tests were conducted under close observation of epilepsy specialists, who did not observe clear‐cut absence seizures, any potential influence of concurrent subclinical epileptiform discharges on performance could not be formally assessed.^48^ We addressed the potential influence of poorly controlled seizures, but could not directly assess the unique effects of GTCS history and/or frequency, owing to the limited sample size and data peculiarities. As expected with a diagnosis of JAE, most individuals (83%) had a history of GTCS, but only six individuals had GTCS in the year prior to the study. ASM can detrimentally affect cognitive performance, particularly topiramate and zonisamide.^49^ For absence epilepsies specifically, attention deficits appear more frequently associated with sodium valproate use than with other ASMs.^50^ Here, some individuals with JAE were taking these medications (one on zonisamide, one on topiramate, 10 [43.5%] on sodium valproate), which may have influenced cognition. Future studies in larger samples are warranted to address the unique influence of specific antiseizure medications and their combinations on cognitive performance in JAE. However, as untreated, unaffected JAE siblings were similarly affected in the language domain, we conclude that such impairment cannot be attributed solely to medication effects.

In conclusion, our study characterizes the cognitive profile of JAE, and identifies wide‐ranging impairment in attention and psychomotor speed, language, and executive function. Linguistic weaknesses cosegregate in patients with JAE and their unaffected siblings, representing familial traits (endophenotypes). The cognitive profiles of JAE and JME largely overlap, but there is evidence of syndrome‐specific impairment in response inhibition. Cognitive abilities, particularly attention/psychomotor speed and executive function, appear to be detrimentally modulated by an early seizure onset and longer disease duration.

## AUTHOR CONTRIBUTIONS

Lorenzo Caciagli, Britta Wandschneider, and Matthias J. Koepp designed the study. Matthias J. Koepp and Britta Wandschneider supervised the study. Lorenzo Caciagli, Maria Centeno, Christian Vollmar, and Britta Wandschneider recruited participants. Lorenzo Caciagli, Britta Wandschneider, Louis A. van Graan, Karin Trimmel, Christian Vollmar, Maria Centeno, and Fenglai Xiao acquired the data. Lorenzo Caciagli, Corey Ratcliffe, and Britta Wandschneider performed the statistical analysis. Lorenzo Caciagli and Britta Wandschneider wrote the paper and revised it based on feedback by Corey Ratcliffe, Fenglai Xiao, Karin Trimmel, Christian Vollmar, Maria Centeno, Louis A. van Graan, Matthias J. Koepp, John S. Duncan, Pamela J. Thompson, and Sallie Baxendale. Lorenzo Caciagli, Matthias J. Koepp, and Britta Wandschneider obtained funding.

## FUNDING INFORMATION

This study was funded by a Henry Smith Charity (20133416) grant awarded to M.J.K. and B.W., by a Wellcome Trust grant (079474) awarded to M.J.K., and by a Brain Research UK scholarship (14181) awarded to L.C. B.W. received salary support from the German Research Foundation (WA3135/1‐1). K.T. was supported by scholarships from the European Academy of Neurology and the Austrian Neurology Society. F.X. is supported by a Newton International Fellowship of the Academy of Medical Sciences and the Newton Fund (NIF\R5\264) and acknowledges support from National Natural Science Foundation of China (82001369), China Postdoctoral Science Foundation (2020M683321), and Post‐Doctor Research Project, West China Hospital, Sichuan University, China (2020HXBH053). This work was supported by the National Institute for Health Research University College London Hospitals Biomedical Research Centre.

## CONFLICT OF INTEREST STATEMENT

None of the authors has any conflict of interest to disclose. We confirm that we have read the Journal's position on issues involved in ethical publication and affirm that this report is consistent with those guidelines.

## PATIENT CONSENT STATEMENT

Written informed consent was obtained from all participants in accordance with the standards of the Declaration of Helsinki.

## CITATION DIVERSITY STATEMENT

Recent work in several fields of science has identified a bias in citation practices such that papers from women and other minority scholars are undercited relative to the number of such papers in the field (https://github.com/dalejn/cleanBib). Here, we sought to proactively consider choosing references that reflect the diversity of the field in thought, form of contribution, gender, race, ethnicity, and other factors. First, we obtained the predicted gender of the first and last author of each reference by using databases that store the probability of a first name being carried by a woman. By this measure (and excluding self‐citations to the first and last authors of our current paper), our references contain 11.63% woman (first)/woman (last), 18.6% man/woman, 23.26% woman/man, and 46.51% man/man. This method is limited in that (1) names, pronouns, and social media profiles used to construct the databases may not, in every case, be indicative of gender identity; and (2) it cannot account for intersex, nonbinary, or transgender people. Second, we obtained predicted racial/ethnic category of the first and last author of each reference by databases that store the probability of a first and last name being carried by an author of color. By this measure (and excluding self‐citations), our references contain 7.77% author of color (first)/author of color (last), 11.05% White author/author of color, 19.95% author of color/White author, and 61.22% White author/White author. This method is limited in that (1) names and Florida voter data to make the predictions may not be indicative of racial/ethnic identity; and (2) it cannot account for Indigenous and mixed‐race authors, or those who may face differential biases due to the ambiguous racialization or ethnicization of their names. We look forward to future work that could help us to better understand how to support equitable practices in science.

## Supporting information


DATA S1

## References

[1] Scheffer IE , Berkovic S , Capovilla G , Connolly MB , French J , Guilhoto L , et al. ILAE classification of the epilepsies: position paper of the ILAE Commission for Classification and Terminology. Epilepsia. 2017;58(4):512–521. 28276062 10.1111/epi.13709 PMC5386840

[2] Loiseau P , Duché B , Pédespan J‐M . Absence epilepsies. Epilepsia. 1995;36(12):1182–1186. 7489694 10.1111/j.1528-1157.1995.tb01060.x

[3] Gardiner M . Genetics of idiopathic generalized epilepsies. Epilepsia. 2005;46(s9):15–20. 10.1111/j.1528-1167.2005.00310.x 16302872

[4] Jernigan TL , Baaré WFC , Stiles J , Madsen KS . Chapter 5—Postnatal brain development: structural imaging of dynamic neurodevelopmental processes. In: Braddick O , Atkinson J , Innocenti GM , editors. Progress in brain research. Amsterdam: Elsevier; 2011. p. 77–92. 10.1016/B978-0-444-53884-0.00019-1 PMC3690327 21489384

[5] Caciagli L , Wandschneider B , Xiao F , Vollmar C , Centeno M , Vos SB , et al. Abnormal hippocampal structure and function in juvenile myoclonic epilepsy and unaffected siblings. Brain. 2019;142(9):2670–2687. 31365054 10.1093/brain/awz215 PMC6776114

[6] Lin JJ , Dabbs K , Riley JD , Jones JE , Jackson DC , Hsu DA , et al. Neurodevelopment in new‐onset juvenile myoclonic epilepsy over the first 2 years. Ann Neurol. 2014;76(5):660–668. 25087843 10.1002/ana.24240 PMC4362677

[7] Wandschneider B , Hong S‐J , Bernhardt BC , Fadaie F , Vollmar C , Koepp MJ , et al. Developmental MRI markers cosegregate juvenile patients with myoclonic epilepsy and their healthy siblings. Neurology. 2019;93(13):e1272–e1280. 31467252 10.1212/WNL.0000000000008173 PMC7011863

[8] Ratcliffe C , Wandschneider B , Baxendale S , Thompson P , Koepp MJ , Caciagli L . Cognitive function in genetic generalized epilepsies: insights from neuropsychology and neuroimaging. Front Neurol. 2020;11:144. 10.3389/fneur.2020.00144 32210904 PMC7076110

[9] Camfield CS , Camfield PR . Juvenile myoclonic epilepsy 25 years after seizure onset. Neurology. 2009;73(13):1041–1045. 19786695 10.1212/WNL.0b013e3181b9c86f

[10] Almane DN , Jones JE , McMillan T , Stafstrom CE , Hsu DA , Seidenberg M , et al. The timing, nature, and range of neurobehavioral comorbidities in juvenile myoclonic epilepsy. Pediatr Neurol. 2019;101:47–52. 31122836 10.1016/j.pediatrneurol.2019.03.011 PMC6752993

[11] Jennum P , Gyllenborg J , Kjellberg J . The social and economic consequences of epilepsy: a controlled national study. Epilepsia. 2011;52(5):949–956. 21275976 10.1111/j.1528-1167.2010.02946.x

[12] Masur D , Shinnar S , Cnaan A , Shinnar RC , Clark P , Wang J , et al. Pretreatment cognitive deficits and treatment effects on attention in childhood absence epilepsy. Neurology. 2013;81(18):1572–1580. 24089388 10.1212/WNL.0b013e3182a9f3ca PMC3806916

[13] Iqbal N , Caswell H , Muir R , Cadden A , Ferguson S , Mackenzie H , et al. Neuropsychological profiles of patients with juvenile myoclonic epilepsy and their siblings: an extended study. Epilepsia. 2015;56(8):1301–1308. 26075864 10.1111/epi.13061

[14] Chowdhury FA , Elwes RDC , Koutroumanidis M , Morris RG , Nashef L , Richardson MP . Impaired cognitive function in idiopathic generalized epilepsy and unaffected family members: an epilepsy endophenotype. Epilepsia. 2014;55(6):835–840. 24702672 10.1111/epi.12604

[15] Wandschneider B , Kopp UA , Kliegel M , Stephani U , Kurlemann G , Janz D , et al. Prospective memory in patients with juvenile myoclonic epilepsy and their healthy siblings. Neurology. 2010;75(24):2161–2167. 21048200 10.1212/WNL.0b013e318202010a

[16] Gottesman II , Gould TD . The endophenotype concept in psychiatry: etymology and strategic intentions. Am J Psychiatry. 2003;160(4):636–645. 12668349 10.1176/appi.ajp.160.4.636

[17] Crunelli V , Lőrincz ML , McCafferty C , Lambert RC , Leresche N , Di Giovanni G , et al. Clinical and experimental insight into pathophysiology, comorbidity and therapy of absence seizures. Brain. 2020;143(8):2341–2368. 32437558 10.1093/brain/awaa072 PMC7447525

[18] Pavone P , Bianchini R , Trifiletti RR , Incorpora G , Pavone A , Parano E . Neuropsychological assessment in children with absence epilepsy. Neurology. 2001;56(8):1047–1051. 11320177 10.1212/wnl.56.8.1047

[19] Jackson DC , Dabbs K , Walker NM , Jones JE , Hsu DA , Stafstrom CE , et al. The neuropsychological and academic substrate of new/recent‐onset epilepsies. J Pediatr. 2013;162(5):1047–1053.e1. 23219245 10.1016/j.jpeds.2012.10.046 PMC3615134

[20] Fonseca Wald ELA , Hendriksen JGM , Drenthen GS , Kuijk SMJV , Aldenkamp AP , Vles JSH , et al. Towards a better understanding of cognitive deficits in absence epilepsy: a systematic review and meta‐analysis. Neuropsychol Rev. 2019;29(4):421–449. 31776780 10.1007/s11065-019-09419-2 PMC6892766

[21] Dharan AL , Bowden SC , Peterson A , Lai A , Seneviratne U , Dabscheck G , et al. A cross‐sectional investigation of cognition and epileptiform discharges in juvenile absence epilepsy. Epilepsia. 2023;64(3):742–753. 36625418 10.1111/epi.17505

[22] Caciagli L , Wandschneider B , Centeno M , Vollmar C , Vos SB , Trimmel K , et al. Motor hyperactivation during cognitive tasks: an endophenotype of juvenile myoclonic epilepsy. Epilepsia. 2020;61(7):1438–1452. 32584424 10.1111/epi.16575 PMC7681252

[23] Zigmond AS , Snaith RP . The hospital anxiety and depression scale. Acta Psychiatr Scand. 1983;67(6):361–370. 6880820 10.1111/j.1600-0447.1983.tb09716.x

[24] Shaw S , Oei TPS , Sawang S . Psychometric validation of the dysexecutive questionnaire (DEX). Psychol Assess. 2015;27(1):138–147. 25602692 10.1037/a0038195

[25] Nelson HE . The national adult reading test (NART): test manual. Windsor: NFER‐Nelson; 1982.

[26] Tombaugh TN . Trail making test A and B: normative data stratified by age and education. Arch Clin Neuropsychol. 2004;19(2):203–214. 15010086 10.1016/S0887-6177(03)00039-8

[27] Homack S , Riccio CA . A meta‐analysis of the sensitivity and specificity of the Stroop Color and Word Test with children. Arch Clin Neuropsychol. 2004;19(6):725–743. 15288327 10.1016/j.acn.2003.09.003

[28] Wechsler D . Wechsler Adult Intelligence Scale—fourth edition (WAIS‐IV). 22nd ed. San Antonio, TX: Pearson; 2008.

[29] Benton AL , Hamsher KD , Sivan AB . Multilingual aphasia examination. 2nd ed. Iowa City, IA: AJA Associates; 1989.

[30] McKenna P , Warrington EK . The graded naming test. Windsor: NEFR‐Nelson; 1983.

[31] Coughlan AK , Hollows SE . The adult memory and information processing battery (AMIPB): test manual. Leeds: A.K. Coughlin, Psychology Dept, St James' Hospital; 1985.

[32] Stern RA , Prohaska ML . Neuropsychological evaluation of executive functioning. In: Dickstein LJ , Riba MB , Oldham JM , editors. Academic psychiatric press review of psychiatry. Washington, DC: American Psychiatric Press; 1996. p. 243–266.

[33] Arrotta K , Reyes A , Kaestner E , McDonald CR , Hermann BP , Barr WB , et al. Cognitive phenotypes in frontal lobe epilepsy. Epilepsia. 2022;63(7):1671–1681. 35429174 10.1111/epi.17260 PMC9545860

[34] Li C . Little's test of missing completely at random. Stata J. 2013;13(4):795–809.

[35] Hermann BP , Zhao Q , Jackson DC , Jones JE , Dabbs K , Almane D , et al. Cognitive phenotypes in childhood idiopathic epilepsies. Epilepsy Behav. 2016;61:269–274. 27442497 10.1016/j.yebeh.2016.05.013 PMC4998056

[36] Guo JN , Kim R , Chen Y , Negishi M , Jhun S , Weiss S , et al. Impaired consciousness in patients with absence seizures investigated by functional MRI, EEG, and behavioural measures: a cross‐sectional study. Lancet Neurol. 2016;15(13):1336–1345. 27839650 10.1016/S1474-4422(16)30295-2 PMC5504428

[37] Killory BD , Bai X , Negishi M , Vega C , Spann MN , Vestal M , et al. Impaired attention and network connectivity in childhood absence epilepsy. Neuroimage. 2011;56(4):2209–2217. 21421063 10.1016/j.neuroimage.2011.03.036 PMC3105167

[38] Wandschneider B , Thompson PJ , Vollmar C , Koepp MJ . Frontal lobe function and structure in juvenile myoclonic epilepsy: a comprehensive review of neuropsychological and imaging data. Epilepsia. 2012;53(12):2091–2098. 23106095 10.1111/epi.12003

[39] Smith A , Syvertsen M , Pal DK . Meta‐analysis of response inhibition in juvenile myoclonic epilepsy. Epilepsy Behav. 2020;106:107038. 10.1016/j.yebeh.2020.107038 32240946

[40] Jackson DC , Jones JE , Hsu DA , Stafstrom CE , Lin JJ , Almane D , et al. Language function in childhood idiopathic epilepsy syndromes. Lang Epilepsy. 2019;193:4–9. 10.1016/j.bandl.2017.12.005 29610055

[41] Marini C , Scheffer IE , Crossland KM , Grinton BE , Phillips FL , McMahon JM , et al. Genetic architecture of idiopathic generalized epilepsy: clinical genetic analysis of 55 multiplex families. Epilepsia. 2004;45(5):467–478. 15101828 10.1111/j.0013-9580.2004.46803.x

[42] Sachdev PS , Blacker D , Blazer DG , Ganguli M , Jeste DV , Paulsen JS , et al. Classifying neurocognitive disorders: the DSM‐5 approach. Nat Rev Neurol. 2014;10(11):634–642. 25266297 10.1038/nrneurol.2014.181

[43] Tondelli M , Vaudano AE , Ruggieri A , Meletti S . Cortical and subcortical brain alterations in juvenile absence epilepsy. NeuroImage Clin. 2016;12:306–311. 27551668 10.1016/j.nicl.2016.07.007 PMC4983643

[44] Tosun D , Siddarth P , Toga AW , Hermann B , Caplan R . Effects of childhood absence epilepsy on associations between regional cortical morphometry and aging and cognitive abilities. Hum Brain Mapp. 2011;32(4):580–591. 21391248 10.1002/hbm.21045 PMC3058615

[45] Drenthen GS , Fonseca Wald ELA , Backes WH , Debeij‐Van Hall MHJA , Hendriksen JGM , Aldenkamp AP , et al. Lower myelin‐water content of the frontal lobe in childhood absence epilepsy. Epilepsia. 2019;60(8):1689–1696. 31283841 10.1111/epi.16280

[46] Ben‐Ari Y , Holmes GL . Effects of seizures on developmental processes in the immature brain. Lancet Neurol. 2006;5(12):1055–1063. 17110286 10.1016/S1474-4422(06)70626-3

[47] Shaw P , Kabani NJ , Lerch JP , Eckstrand K , Lenroot R , Gogtay N , et al. Neurodevelopmental trajectories of the human cerebral cortex. J Neurosci. 2008;28(14):3586–3594. 18385317 10.1523/JNEUROSCI.5309-07.2008 PMC6671079

[48] Binnie CD . Cognitive impairment during epileptiform discharges: is it ever justifiable to treat the EEG? Lancet Neurol. 2003;2(12):725–730. 14636777 10.1016/s1474-4422(03)00584-2

[49] Wandschneider B , Burdett J , Townsend L , Hill A , Thompson PJ , Duncan JS , et al. Effect of topiramate and zonisamide on fMRI cognitive networks. Neurology. 2017;88(12):1165–1171. 28213372 10.1212/WNL.0000000000003736 PMC5373787

[50] Glauser TA , Cnaan A , Shinnar S , Hirtz DG , Dlugos D , Masur D , et al. Ethosuximide, valproic acid, and lamotrigine in childhood absence epilepsy: initial monotherapy outcomes at 12 months. Epilepsia. 2013;54(1):141–155. 23167925 10.1111/epi.12028 PMC3538883

