# Supplementary material for: Cognitive phenotype of juvenile absence epilepsy: An investigation of patients and unaffected siblings
Source: Epilepsia. 2023 Aug 10;64(10):2792–805. doi: 10.1111/epi.17719 (PMC10952612; doi:10.1111/epi.17719)
Supplement: Supplementary file 1 — DATA S1 [file EPI-64-2792-s001.docx]

***Supplementary material for:***

**The cognitive phenotype of juvenile absence epilepsy and its heritability: An investigation of patients and unaffected siblings**

Lorenzo Caciagli^1,2,3^, Corey Ratcliffe^1,2,4,5^, Fenglai Xiao^1,2^, Louis A. van Graan^1,2^, Karin Trimmel^1,2,6^, Christian Vollmar^1,2,7^, Maria Centeno^1,2,8^, John S. Duncan^1,2^, Pamela J. Thompson^1,2^, Sallie Baxendale ^1,2^, Matthias J. Koepp ^1,2^*****, Britta Wandschneider^1,2^*****

***** *Equal contribution as senior author*

1. *Department of Clinical and Experimental Epilepsy, UCL Queen Square Institute of Neurology, London WC1N 3BG, United Kingdom*
2. *MRI Unit, Epilepsy Society, Chalfont St Peter, Buckinghamshire, SL9 0RJ United Kingdom*
3. *Department of Neurology, Inselspital, Sleep-Wake-Epilepsy-Center, Bern University Hospital, and University of Bern, Bern, Switzerland*
4. *Department of Pharmacology and Therapeutics, Institute of Systems, Molecular, and Integrative Biology, University of Liverpool, Liverpool, UK*
5. *Department of Neuroimaging and Interventional Radiology, National Institute of Mental Health and Neurosciences, Bangalore, India*
6. *Department of Neurology, Medical University of Vienna, Vienna, Austria*
7. *Department of Neurology, Ludwig-Maximilians-Universität, 81377 Munich, Germany*
8. *Epilepsy Unit, Department of Neurology, Hospital Clínic de Barcelona, 08036 Barcelona, Spain*

**Corresponding Author**

Dr Britta Wandschneider, MD PhD

UCL Queen Square Institute of Neurology

Department of Clinical and Experimental Epilepsy

London WC1N 3BG, United Kingdom

E-mail: b.wandschneider@ucl.ac.uk

**SUPPLEMENTARY METHODS**

*Assignment of cognitive tests to cognitive domains*

To ascertain whether letter fluency ought to be assigned to the language or executive function domain, we assessed correlations between letter fluency and (i) other language measures (vocabulary, naming, and semantic fluency), and (ii) other executive function measures [digit span, arithmetic, Trail Making Test (TMT) B-A, Stroop– Interference] across all study participants. For language measures, there were significant correlations between letter fluency and vocabulary (*r*= 0.40, *p*<.001), naming (*r*= 0.25, *p*=.009) and semantic fluency (*r*= 0.49, *p*<.001). On the other hand, letter fluency was significantly correlated with TMT B-A (*r*= -0.28, *p*=.002), digit span (*r*= 0.31, *p*=.001), arithmetic (*r*= 0.35, *p*<.001), but not Stroop– Interference scores (*r*= 0.08, *p*=.44). Consequently, letter fluency was included in the language domain. A similar approach was adopted for the similarities subtest of the WAIS. For language measures, there were significant correlations between similarities and vocabulary (*r*= 0.71, *p*<.001), naming (*r*= 0.34, *p*<.001) and semantic fluency (*r*= 0.35, *p*<.001). Similarities scores were also significantly correlated with TMT B-A (*r*= -0.44, *p*<.001), digit span (*r*= 0.24, *p*=.01), arithmetic (*r*= 0.69, *p*<.001), but not with Stroop interference scores (*r*= -0.13, *p*=.21). Thus, the Similarities subtest of the WAIS was also included in the language domain.

*Norm-based analyses: illustration of selected norms and effect size comparisons*

Analyses using neuropsychological test norms contextualized group differences between JAE and controls from a clinical standpoint. Norm-based analyses focused on domains and test for which differences between individuals with JAE and controls were statistically significant: (i) attention/psychomotor speed, (ii) language, and (iii) executive function. The following references were used:

- *Attention/psychomotor speed:* Updated *Golden* norms^1^ for the Word and Color scores of the Stroop Test; *Tombaugh* norms^2^ for the TMT– Part A.
- *Language*: Wechsler Adult Intelligence Scale (WAIS) III edition for the Vocabulary and Similarities subtests;^3^ British, London-based norms for the McKenna Graded Naming test.^4^
- *Executive function:* Wechsler Adult Intelligence Scale (WAIS) III edition for the Digit Span and Arithmetic subtests.^3^

No suitable norms were found for the following measures:

1. TMT B-A: the Tombaugh norms^2^ provide normative references only for TMT– Part A and Part B scores.
2. British norms for letter fluency F, A, and S sum scores: in this case, British and geographically representative (London-based) norms^4^ were considered more suitable than other Controlled Oral Word Association Test norms.
3. British norms for all the three categories used in our category fluency test (Animals, Fruits, Vegetables).
4. Stroop– Interference score (The updated Goldman norms compute an interference score that is different from the one used in our center).

We therefore validated the appropriateness of surrogate norms for the above four cases, as follows: (i) *Tombaugh* norms for TMT, Part B scores^2^ instead of TMT B-A scores; (ii) British, London-based test norms^4^ for letter fluency (letter “S”) and (iii) category fluency (“Animals”); (iv) Updated *Golden* norms^1^ for the Word-Color scores of the Stroop Test. To assess appropriateness replacement, we verified that effect sizes relating to group differences between individuals with JAE and controls for the original and surrogate measure were comparable using *Z*-tests, as follows:^5^

$$z_{\mathrm{diff}}= \frac{d_{1}-d_{2}}{\sqrt{\upsilon_{1}+\upsilon_{2}}},$$

Where *d_1_*/*υ*_1_ and *d_2_*/*υ*_2_ stand for Cohen’s *d*/its sampling variance for the original and surrogate measures, respectively. For |𝑧_diff_| ≤ 2.5, there is no differences between effect sizes at a threshold of p ⪅ 0.05 (two-tailed, Bonferroni-corrected across 4 tests) and the surrogate measure can thus be considered as representative.

We obtained the following results: (i) Cohen’s *d =* -1.38 and -1.16 for TMT– B and TMT– B-A, respectively, *z*= |1.57|; (ii) Cohen’s *d=* -0.85 and -1.12, *z*=|2.22| for letter S fluency and FAS fluency scores; (iii) Cohen’s *d=* -1.05 and -1.03 for category fluency, Animals only and Animals/Fruits/Vegetables scores, *z*= |0.13|; (iv) Cohen’s *d=* 0.99 and 0.27, Stroop– Color-Word and Stroop Interference scores, *z*=|5.61|. Thus, we considered the substitute TMT–B, letter S fluency, and Animal naming norms as appropriate surrogates, while Stroop Word-Color were not considered a suitable surrogate of the Stroop Interference Score.

**SUPPLEMENTARY RESULTS**

*MANCOVA on JAE, JAE siblings and controls: sensitivity analyses*

Repeat MANCOVA covarying for *education* in addition to age and sex confirmed a significant effect of group on cognitive performance (Wilk's λ=.57, *F*_(10,84)_=2.7, *p*=.006). There were no significant effects of education neither at the multivariate level nor in each individual cognitive domain. Covarying for education did not affect the statistical significance of group differences for attention/psychomotor speed (JAE vs controls: *p*_Bonferroni_*=*.009), language (JAE vs controls: *p*_Bonferroni_ *<*.001; siblings vs controls: *p*_Bonferroni_*=*.006), and global executive function performance (JAE vs controls: *p*_Bonferroni_ *<*.001).

Repeat MANCOVA covarying for self-reported *anxiety* and *depression* symptoms in addition to age and sex confirmed a significant effect of group on cognitive performance (Wilk’s λ=.57, *F*_(10_*_,_*_82)_=2.7, *p*=.019). There were no significant effects of anxiety and depression at the multivariate level nor in each individual cognitive domain. Covarying for anxiety and depression did not affect the statistical significance of group differences for attention/psychomotor speed (JAE vs controls: *p*_Bonferroni_*=*.012), language (JAE and siblings vs controls: both *p*_Bonferroni_ *<*.001), and global executive function performance (JAE vs controls: *p*_Bonferroni_ *<*.001).

*MANCOVA on JME versus controls*

There was a significant effect of group on cognitive performance (Wilk’s λ= .56, *F*_(5,42)_=6.5, *p*=.0001). There were no significant differences in IQ between individuals with JME and controls. Individuals with JME performed worse than controls across domains of language, global executive function and response inhibition-weighted executive function (all *p*_FDR_ ≤.01; *d* range: -1.06 to -.85; Supplementary Table 6).

**SUPPLEMENTARY TABLE 1. Cognitive tests.**

| Domain | Test | Test description |
| --- | --- | --- |
| *General intellectual abilities* | *NART* | Participants are asked to read 50 irregularly spelled and/or pronounced British English words. Measure of estimated IQ. |
| *Attention/Psychomotor Speed* | *Trail Making Test:*  *Time – Part A* | Participants are asked to connect numbers in ascending order, using a continuous line, as quickly as possible. |
|  | *Stroop– Color (C)* | Participants are asked to read a list of named ink colors, and the maximum number of words read within 45 seconds is recorded. |
|  | *Stroop– Word (W)* | Participants are asked to read a list of color words, and the maximum number of words read within 45 seconds is recorded. |
| *Language* | *Vocabulary (WAIS III)* | Participants are required to provide definitions for 33 specific words of increasing difficulties. |
|  | *Similarities (WAIS III)* | Participants are asked to describe the relationship (common link) between 19 pairs of words. |
|  | *Controlled Oral Word Association Test* | Participants are asked to name words starting with a specific letter (F, A, or S) in one minute. Probes letter fluency. |
|  | *Category fluency test* | Participants are asked to name items subsumed under a specific category in one minute; in our study, the three tested categories were: animals, fruits, and vegetables. |
|  | *McKenna Graded Naming test* | Participants are asked to name 30 items of graded difficulty that are depicted as black-and-white line drawings. |
| *Memory* | *AMIPB: List Learning* | Participants are asked to memorize a 15-item word list over five trials (List A1-A5) |
|  | *AMIPB: List Recall* | Participants are asked to recall the original word list, memorized during the learning phase of the test, following a distracting list. |
|  | *AMIPB: Design Learning* | Participants are asked to reproduce a 9-element design on a 4 × 4 grid over five consecutive trials (Design A1-A5). |
|  | *AMIPB: Design Recall* | Participants are asked to reproduce the original design, memorized during the learning phase of the test, following a distracting design |
| *Executive function* | *Digit Span* | Participants are asked to repeat a set of numbers of increasing length in the correct order upon presentation; then, they are asked to repeat second set of numbers in reverse order. Probes working memory. |
|  | *Arithmetic* | Participants are asked to solve orally presented arithmetic problems without using pen and paper. Probes working memory. |
|  | *Trail Making Test:*  *Task Switching (B-A)* | Participants are asked to connect numbers and letters of the alphabet in sequence, alternating between letters and numbers, as fast as possible. Probes cognitive flexibility. |
|  | *Stroop – Interference* | Colored Words condition: participants are asked to name the ink color of color words that written with an incongruent color as quickly as possible. Formula: Interference= \|CW − [(W + C)/2]\|. Probes response inhibition. |

*Abbreviations.* AMIPB= Adult Memory and Information Processing Battery; NART= National Adult Reading Test; WAIS= Wechsler Adult Intelligence Scale. Test references are provided in the main manuscript text.

**SUPPLEMENTARY TABLE 2. PCA, Attention/Psychomotor Speed**

| Attention/psychomotor speed. Eigenvalue= 1.85; Explained variance= 61.7% | | | |
| --- | --- | --- | --- |
| *Correlation matrix – cognitive tests subsumed under domain* | | | |
|  | Trail Making Test (Part A) | Stroop– Words | Stroop– Color |
| Trail Making Test (Part A) | 1.00 |  |  |
| Stroop– Word | -.33  (<.001) | 1.00 |  |
| Stroop– Color | -.26  (.009) | .64  (<.001) | 1.00 |
| *Component Loadings* | -.601 | .878 | .847 |

The table reports: (1) the correlation matrix pertaining to tests included in the principal component analysis (PCA), whereby a given cell reports the correlation coefficient (product-moment correlation) and associated *p-*value for a given test pair; and (2) the component loadings for each variable entered in the PCA.

**SUPPLEMENTARY TABLE 3. PCA, Language**

| Language. Eigenvalue= 2.63; Explained variance= 52.5% | | | | | |
| --- | --- | --- | --- | --- | --- |
| *Correlation matrix – cognitive tests subsumed under domain* | | | | | |
|  | Vocabulary | Similarities | Letter  fluency | Category fluency | McKenna Graded Naming |
| Vocabulary | 1.00 |  |  |  |  |
| Similarities | .70  (<.001) | 1.00  (<.001) |  |  |  |
| Letter fluency | .39  (<.001) | .32  (<.001) | 1.00 |  |  |
| Category fluency | .39  (<.001) | .33  (<.001) | .49  (<.001) | 1.00 |  |
| McKenna Graded Naming | .51  (<.001) | .34  (<.001) | .21  (.030) | .30  (.002) | 1.00 |
| *Component Loadings* | .86 | .77 | .65 | .68 | .64 |

The table reports: (1) the correlation matrix pertaining to tests included in the principal component analysis (PCA), whereby a a given cell reports the correlation coefficient (product-moment correlation) and associated *p-*value for a given test pair; and (2) the component loadings for each variable entered in the PCA.

**SUPPLEMENTARY TABLE 4. PCA, Memory**

| Memory. Eigenvalue= 2.37; Explained variance= 59.1% | | | | |
| --- | --- | --- | --- | --- |
| *Correlation matrix – cognitive tests subsumed under domain* | | | | |
|  | List A1-5 | List A6 | Design A1-5 | Design A6 |
| List A1-5 | 1.00 |  |  |  |
| List A6 | .75  (<.001) | 1.00 |  |  |
| Design A1-5 | .33  (<.001) | .36  (<.001) | 1.00 |  |
| Design A6 | .27  (.005) | .26  (.006) | .75  (<.001) | 1.00 |
| *Component Loadings* | .86 | .77 | .65 | .68 |

The table reports: (1) the correlation matrix pertaining to tests included in the principal component analysis (PCA), whereby a given cell reports the correlation coefficient (product-moment correlation) and associated *p-*value for a given test pair; and (2) the component loadings for each variable entered in the PCA.

**SUPPLEMENTARY TABLE 5. PCA, Executive function**

| Executive function 1 (EF1). Eigenvalue= 1.96; Explained variance= 49.0% | | | | |
| --- | --- | --- | --- | --- |
| Executive function 2 (EF2). Eigenvalue= 1.03; Explained variance= 25.7% | | | | |
| *Correlation matrix – cognitive tests subsumed under domain* | | | | |
|  | Trail Making Test (B-A) | Digit Span | Arithmetic | Stroop– Interference |
| Trail Making Test (B-A) | 1.00 |  |  |  |
| Digit Span | -.40  (<.001) | 1.00 |  |  |
| Arithmetic | -.54  (<.001) | .43  (<.001) | 1.00 |  |
| Stroop– Interference | .12  (.26) | .02  (.85) | -.25  (.02) | 1.00 |
| *Component Loadings (EF1)* | -.81 | .70 | .85 | -.31 |
| *Component Loadings (EF2)* | -.09 | .43 | -.10 | .91 |

The table reports: (1) the correlation matrix pertaining to tests included in the principal component analysis (PCA), whereby a given cell reports the correlation coefficient (product-moment correlation) and associated *p-*value for a given test pair; and (2) the component loadings for each variable entered in the PCA.

**SUPPLEMENTARY TABLE 6.** **Comparison of JME and controls.**

| *Multivariate model*: Wilk’s Lambda= .56, *F*_(5,42)_=6.5, *p*= .0001 | | | | |
| --- | --- | --- | --- | --- |
|  | **Effect of group**  **(*F* statistic)** | ***P_FDR_* value**  **(uncorr. *P*)** | **Mean (SD)** | **Effect size**  **(Cohen’s *d*)** |
| Estimated IQ  (NART) | F_1,72_ = .37 | .544  (.544) | JME: 108.4 (10.9)  CTR: 108.8 (7.7) | JME vs CTR: -.13 |
| Attention/ psychomotor speed | F_1,62_ = 2.5 | .139  (.116) | JME: -.08 (1.2)  CTR: .40 (.76) | JME vs CTR: -.37 |
| Language | F_1,65_ = 23.9 | **.0004**  **(<.0001)** | JME: -.11 (.96)  CTR: .69 (.65) | JME vs CTR: **-1.06** |
| Memory | F_1,72_ = 3.7 | .085  **(**.057**)** | JME: -.29 (.98)  CTR: .34 (.84) | JME vs CTR: -.42 |
| Executive function (*global*) | F_1,51_ = 12.9 | **.002**  **(.0007)** | JME: -.15 (1.1)  CTR: .47 (.69) | JME vs CTR: **-.85** |
| Executive function  (*response inhibition*) | F_1,51_ = 14.1 | **.001**  (**.0004**) | JME: -.81 (1.00)  CTR: .35 (.68) | JME vs CTR: **-.89** |

Abbreviations. CTR= healthy controls; JME= Juvenile Myoclonic Epilepsy; NART= National Adult Reading Test; SD= standard deviation; uncorr.= uncorrected. All statistical analyses controlled for age and sex.

**SUPPLEMENTARY FIGURE 1. JAE, siblings, controls: IQ**

*
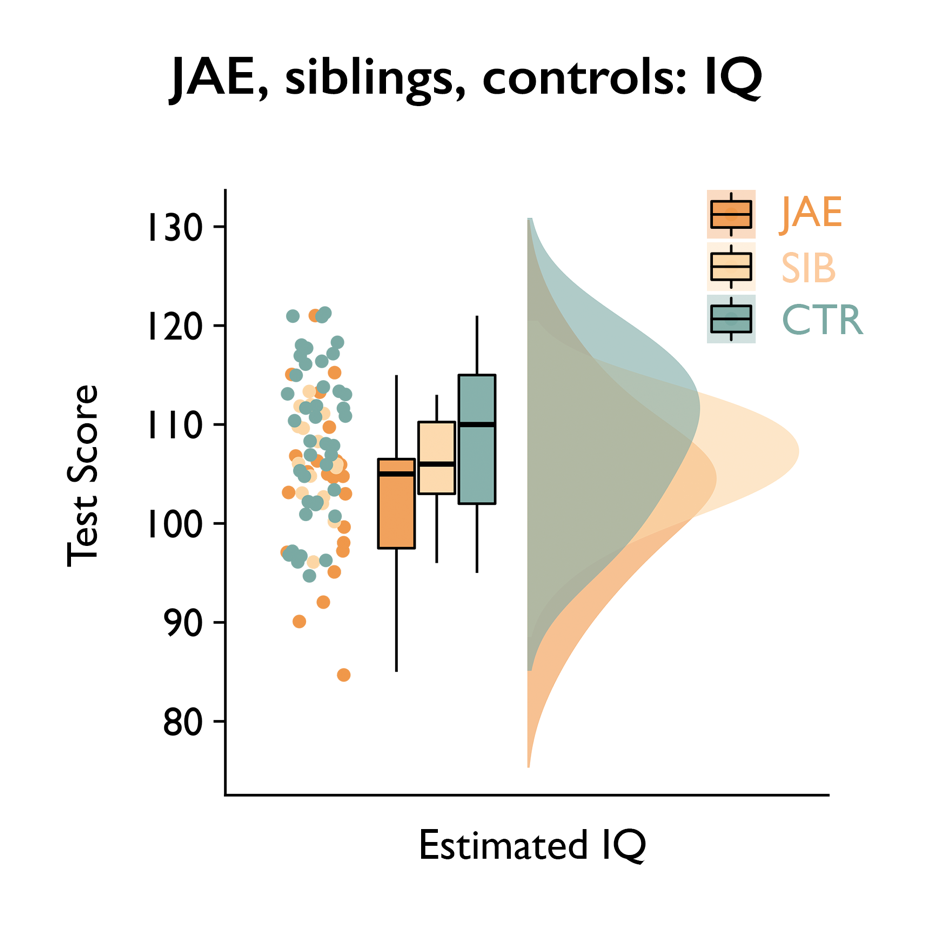
*

The figure shows data pertaining to estimated IQ scores (National Adult Reading Test) in individuals with JAE, unaffected JAE siblings (SIB) and controls (CTR). We used open-source code to generate *raincloud* plots (https://github.com/RainCloudPlots/RainCloudPlots) and show a combination of single datapoints (raw scores), boxplots, and probability distributions. Statistical details are reported in Table 2 and in the main manuscript text.

**SUPPLEMENTARY REFERENCES**

1. Golden CJ, Freshwater, S.M., Syzdek, B.M., Ailes, E.L. Stroop Color and Word Test Normative Update.: Stoelting; 2021.

2. Tombaugh TN. Trail Making Test A and B: normative data stratified by age and education Arch Clin Neuropsychol. 2004 Mar;19:203-214.

3. Wechsler D. Wechsler adult intelligence scale—3rd ed.(WAIS-3®). San Antonio, TX: Harcourt Assessment; 1997.

4. Bird CM, Papadopoulou K, Ricciardelli P, Rossor MN, Cipolotti L. Monitoring cognitive changes: psychometric properties of six cognitive tests Br J Clin Psychol. 2004 Jun;43:197-210.

5. Borenstein M, Hedges, L.V., Higgins, J.P.T., Rothstein, H.R. Introduction to Meta-Analysis. John Wiley & Sons L, editor2009.
